# Red blood cell transfusion in patients with traumatic brain injury: a systematic review protocol

**DOI:** 10.1186/2046-4053-3-66

**Published:** 2014-06-18

**Authors:** Amélie Boutin, Michaël Chassé, Michèle Shemilt, François Lauzier, Lynne Moore, Ryan Zarychanski, Jacques Lacroix, Dean A Fergusson, Philippe Desjardins, Alexis F Turgeon

**Affiliations:** 1 Department of Social and Preventive Medicine, Université Laval, Québec, QC, Canada; 2 Centre Hospitalier Universitaire (CHU) de Québec Research Center, Population Health and Optimal Health Practices Research Unit, Traumatology-Emergency-Critical Care Medicine, Université Laval, Québec, QC, Canada; 3 Division of Critical Care Medicine, Department of Anesthesiology and Critical Care Medicine, Université Laval, Québec, QC, Canada; 4 Department of Medicine, Université Laval, Québec, QC, Canada; 5 Department of Internal Medicine, Sections of Critical Care Medicine of Hematology and of Medical Oncology, University of Manitoba, Winnipeg, MB, Canada; 6 Department of Pediatrics, Critical Care Medicine, Université de Montréal, Montréal, QC, Canada; 7 Clinical Epidemiology Unit, Ottawa Hospital Research Institute, Ottawa, ON, Canada

**Keywords:** Red blood cells transfusion, Blood products, Traumatic brain injuries

## Abstract

**Background:**

Anemia is a prevalent condition in critically ill patients and red blood cell transfusions are frequent. Although transfusions at low hemoglobin levels have been shown to be associated with equivalent or better outcomes than higher hemoglobin thresholds, clinical equipoise persists in patients with traumatic brain injury considering their susceptibility to secondary cerebral insults such as those from hypoxemia.

**Methods:**

Our objectives are to estimate the frequency of red blood cell transfusion in patients with traumatic brain injury and to evaluate transfusion thresholds, determinants and outcomes associated with transfusion strategies.

We will conduct a systematic review of cohort studies and randomized controlled trials of patients with traumatic brain injury. We will search MEDLINE, Embase, BIOSIS and the Cochrane Library for eligible studies. Two independent reviewers will screen all identified references. Studies including adult patients with traumatic brain injury reporting data on red blood cell transfusions will be eligible. We will collect data on baseline demographics, trauma characteristics, hemoglobin thresholds, blood transfusions and clinical outcomes (mortality, length of stay, complications, and so on). Two independent reviewers will extract data using a standardized form. We will pool cumulative incidences using DerSimonian and Lair random-effect models after a Freeman-Tukey transformation to stabilize variances. We will pool risk ratios or mean differences with random-effect models and Mantel-Haenszel or inverse variance methods in order to evaluate the association between red blood cell transfusion and potential determinants or outcomes. Sensitivity and subgroup analysis according to timing of red blood cell transfusion, traumatic brain injury severity, year of conduction of the study, risk of bias, notably, are planned.

**Discussion:**

We expect to observe high heterogeneity in the proportion of transfused patients across studies and that the global proportion will be similar to the frequency observed in the general medical critically ill population. Our systematic review will allow us to better describe and understand current transfusion practices in patients with traumatic brain injury, a clinical population in which liberal transfusions are still advocated in the absence of evidence-based data.

**Systematic review registration:**

PROSPERO: CRD42014007402.

## Background

Red blood cell transfusion is a core topic in critical care medicine. Transfusion practices in the overall non-bleeding medical and surgical intensive care population have been extensively described [1,2]. Studies have evaluated hemoglobin thresholds for transfusion in critically ill patients [3-13] and have shown that restrictive transfusion strategies (hemoglobin thresholds between 7 and 9 g/dL) are as safe as liberal strategies (thresholds between 9 and 12 g/dL) [3,4]. However, specific patient populations, such as neurocritically ill patients, were underrepresented in these studies and results could thus not be applied to them. Indeed, given the vulnerability of the brain to secondary hypoxic insults, concerns have been raised regarding the safety and efficacy of restrictive transfusion strategies in the presence of traumatic brain injuries [14]. Two recent guidelines in a neurocritically ill patient population (subarachnoid hemorrhage) were published; one recommending to treat anemia but noting that thresholds were to be determined, and the other recommending transfusion in order to reach hemoglobin levels of 80 to 100 g/L [15,16]. Interestingly, guidelines for the management of patients with traumatic brain injury did not cover the topic [17]. A recent systematic review highlighted the paucity of data regarding the adoption of liberal or restrictive strategies in this specific population [18] and no consensus has been reached on appropriate transfusion thresholds [19]. Considering the high mortality in critically ill patients with traumatic brain injury [20], the potential impact of red blood cells transfusion on clinical outcomes and the uncertainty regarding optimal transfusion strategies in patients with acute neurologic lesions, current transfusion practices must be described in order to inform future clinical trials evaluating transfusion strategies in this population.

### Objectives

We first aim to evaluate current practices regarding red blood cell transfusion in critically ill patients with traumatic brain injuries by estimating the frequency of red blood cell transfusion in these patients. Secondly, we seek to evaluate transfusion thresholds, determinants and outcomes associated with transfusion strategies.

## Methods

We propose to conduct a systematic review of cohort studies and randomized control trials reporting transfusions in patients with traumatic brain injury during their acute hospital stay.

### Protocol and registration

The protocol of the review is registered in PROSPERO (http://www.crd.york.ac.uk/prospero) CRD42014007402.

### Study design

We will conduct a systematic review in accordance with the Preferred Reporting Items for Systematic Reviews and Meta-Analyses (PRISMA) statement [21] and The Cochrane Handbook for Systematic Reviews of Interventions [22] methodological recommendations.

### Eligibility criteria

Since we are interested in the frequency of red blood cell transfusion and its determinants, our systematic review will include prospective and retrospective cohort studies, and randomized controlled trials. Patients suffering from a traumatic brain lesion (any severity) will be considered. In case of a mixed population, at least 80% of patients included in a specific study have to respect this criterion for the study to be eligible. Since we expect that few studies will report precisely if patients were recruited at hospital admission or intensive care unit (ICU) admission, we will not restrict inclusion to critically ill patients but rather consider a population-based approach of acute care hospital admissions. Studies and trials will have to report data on red blood cell transfusion frequency. Studies who specifically studied patients with blood disorders and coagulopathies will be excluded. Table 1 and Table 2 present the structured study question and inclusion and exclusion criteria, respectively.

**Table 1:** Structured question

Population	• adult patients with traumatic brain injury
Intervention	• red blood cell transfusion
Comparator	• no transfusion
Primary outcome	• frequency of red blood cell transfusion
Secondary outcomes	• transfusion thresholds
	• number of red blood cell units transfused
	• determinants of red blood cell transfusion
	• mortality
	• frequency of withdrawal of life-sustaining therapy
	• frequency of unfavourable neurological outcome
	• ICU and hospital length of stay
	• all other reported clinical outcomes
Study design	• cohort studies (both prospective and retrospective) and randomized controlled trials

**Table 2:** Study eligibility criteria

Inclusion criteria	• Prospective study, retrospective cohort study or randomized controlled trials
	• Acute setting
	• At least 80% of patients suffering from an *acute TRAUMATIC brain lesion*
	• At least 80% of adults patients (≥18 years old)
	• Data on red blood cell transfusion reported
Exclusion criteria	• Sample of patients with congenital hereditary blood disorders (example: sickle cell disease, β-thalassemia)
	• Sample of patients with coagulation disorders (example: hemophilia, thrombotic thrombocytopenic purpura, Von Willebrand disease)

### Information sources

We will systematically search MEDLINE, Embase, BIOSIS and The Cochrane Library (from their inception up to a maximum of nine months before submission for publication) for eligible studies. References of included articles and abstracts of major conferences will be screened to identify additional potentially eligible studies. Experts in neurocritical care medicine, not members of our team, will also be contacted to identify additional ongoing studies. We will request available transfusion data from investigators of retrieved studies if deemed necessary.

### Search strategy

Our search strategy will be based on keywords related to transfusion and anemia, as well as traumatic brain injury. Clinicians, investigators with expertise in transfusion or in neurocritical care, and information specialists will be consulted to verify the search strategy, identify synonyms and additional search terms. Relevant index terms (Medical Subject Headings and Emtree) will be added to the strategy. The search will be limited to human studies [22]. No language or date of publication restriction will be used. The search strategy will be first designed for Medline and Embase, and will be adapted for other electronic databases afterwards. The current version of our Medline search strategy is presented in Additional file 1. This preliminary strategy has been tested through an iterative process in order to achieve sufficient specificity while maintaining high sensitivity. Results will be imported in EndNote (version X7.0.1, New York City: Thomson Reuters, 2011) and duplicates will be removed. References will then be exported to a Microsoft Excel (version 14.1.0, Redmond, WA: Microsoft, 2011) spreadsheet in order to complete the selection process.

### Study selection

Two independent reviewers will screen all identified references to determine eligibility, first from titles and abstracts, and then based on full text evaluation for studies that could be potentially eligible. In case of disagreement on the inclusion of a study, a third reviewer will be consulted. In case the blood product transfused is not clearly specified, authors will be contacted to ensure that reported data pertains to red blood cell transfusion.

A translation of non-English or non-French articles will be obtained. Agreement on study selection will be evaluated with a kappa coefficient. Considering the high sensitivity of the search strategy, we expect the kappa will indicate moderate agreement. In case the agreement is too low, indicating an evasive interpretation of eligibility criteria, a third reviewer will review records’ titles and abstracts.

### Data collection process

A preliminary version of the abstraction form will be pilot-tested and customized by two reviewers using four publications. Two independent reviewers will abstract data using the standardized form. In case of discrepancy, consensus will be reached with the involvement of a third reviewer. Authors will be contacted if relevant data is missing or clarification is needed.

### Data items

Data pertaining to study characteristics (design, date of completion, funding sources, and so on), patients’ baseline characteristics (age, gender, type of neurological disease, severity of the lesion on admission, and so on), clinical management (surgical, medical), hemoglobin levels, blood products (type of products received, timing, quantity, repetition, thresholds, and so on), co-interventions (type, timing), clinical outcomes (mortality and withdrawal of life-sustaining therapies and their timing, length of hospital and ICU stay, neurological outcome (any scale; for example, Glasgow Outcome Score (GOS)), complications, and so on) [Additional file 2] will be extracted from published reports.

### Risk of bias in individual studies

Risk of bias of included RCTs will be assessed using The Cochrane Collaboration tool for assessing the risk of bias [22]. Risk of bias in cohort studies will be assessed using a pilot version of a new tool for the assessment of the risk of bias in non-randomized studies, currently under development by Cochrane’s Non-Randomized Studies Methods Group.

### Summary measures

We will report the cumulative incidence of transfusion in the course of hospital stay (primary outcome). Risk ratios of the association between red blood cell transfusion and potential determinants (categorical variables such as sex) or any relevant clinical outcomes, such as mortality, unfavorable neurological outcome, complications (any or specific complications, or categories of complications [23]), will be reported. Mean differences of potential determinants or outcomes (continuous or ordinal variables such as age, hemoglobin levels, severity of the traumatic brain injury, length of stay, and so on) according to transfusion status will be reported. We will compute mean transfusion thresholds as well as mean differences in number of units transfused according to outcomes when available. A two-sided 5% type I error will be considered for all analyses.

### Synthesis of results

If appropriate, results from cohort studies will be pooled with results from randomized controlled trials that did not randomize patients to specific transfusion strategies. Results from randomized controlled trials allocating patients to different transfusion strategies will be pooled separately, if deemed appropriate.

Variances of cumulative incidences of transfusion from all studies will be stabilized using a Freeman-Tukey transformation [24] and proportions will be pooled with DerSimonian and Laird random-effects approach [25] using R statistical software (version 2.15.1: R Core Team, R Foundation for Statistical Computing June 2012, Vienna, Austria). Means and mean differences will be pooled with inverse variance method with random effects. Risk ratio analyses will be conducted with Review Manager (RevMan) (version 5.1, Copenhagen: The Nordic Cochrane Centre, The Cochrane Collaboration, 2012) using Mantel-Haenszel random-effect models. Pooled effect sizes and their 95% confidence limits will be reported.

Statistical heterogeneity will be measured using the Cochran’s *Q*-test and I^2^ statistics [26], the latter being interpreted as low from 0 to 40%, moderate from 30 to 60%, substantial from 50 to 90% and considerable from 75 to 100% [22].

### Risk of bias

We will evaluate the risk of publication bias by visual exploration of funnel plots. We will also evaluate the risk of selective reporting of outcomes within studies by searching for previously published protocols on registration website (http://www.controlled-trials.com and clinicaltrials.gov).

The ability of a study to answer the review question will be evaluated in terms of applicability. Applicability concerns relate to deviation of a study from the ideal study designed to answer our research question (in relation to our primary outcome). For instance, a study recruiting patients with any severity of traumatic brain injury at hospital admission, therefore including patients with mild traumatic brain injury that might not be admitted to an ICU, will be considered as having high applicability concerns.

### Additional analyses

#### ***Sensitivity analyses***

To assess the strength of observed associations, *a priori* sensitivity analyses are planned to explore potential heterogeneity according to the following factors: severity of the brain injury (moderate or severe traumatic brain injury; severe only, defined as Glasgow Coma Scale (GCS) < 9 or according), type of blood product given as a co-intervention (platelets, plasma, whole blood, and so on), risk of bias (low risk of bias), low applicability concerns, design of studies (cohort versus randomized controlled trials), presence of comorbidities (by categories of comorbidities [27] if data available) and year of publication (after 1999, year of the TRICC trial publication [5]). A sensitivity analysis will also be conducted taking into account if outcomes were reported as primary or secondary outcomes.

#### ***Subgroup analyses***

Subgroup analyses to assess clinical heterogeneity are also planned to evaluate timing of intervention (emergency, ICU, overall hospital stay or other timing), time spent in the ICU or hospital, amount of blood transfused, surgery or specific pharmacological interventions, volume replacement, active bleeding and CRASH and IMPACT scores.

#### ***Meta-regression***

If the number of eligible studies is sufficient (at least ten studies by covariate), we also plan on conducting a meta-regression analysis; first, modeling mean values of multiple determinants at study level with the reception of a transfusion; secondly, transfusion and factors associated with mortality in traumatic brain injury (for example, age and GCS) with mortality.

#### ***GRADE of evidences***

We will use the GRADE methodology to evaluate the quality of evidences (http://www.gradeworkinggroup.org) of our findings [28].

## Discussion

### Expected benefits

This project will allow the knowledge synthesis regarding transfusion practices in patients with traumatic brain injury. Considering the paucity of data and the equipoise on the optimal transfusion strategy in this population, it is of major importance to assess current practices. Therefore, this project, in addition to our previous systematic review of comparative studies, will systematically group original research data on the topic of red blood cell transfusion in traumatic brain injury.

### Inform future studies

Considering the observed lack of evidence from comparative studies and the risk of bias associated with observational studies, we expect results to be heterogeneous. Our results will provide information to inform the design of further studies in traumatic brain injury and red blood cells transfusion. Ultimately, we will obtain critical data regarding transfusion practices in patients with traumatic brain injury that will allow us to better design a high-quality non-inferiority trial. For example, information on the frequency of transfusion among patients with traumatic brain injury will facilitate the calculation of sample size estimates and recruitment rates. The knowledge of the usual hemoglobin thresholds observed in patients with traumatic brain injury and those associated with better outcomes will help setting acceptable, plausible and realistic comparative thresholds in a future trial.

### Limitations

Despite the use of rigorous methodology, we do expect high statistical and clinical heterogeneity in our analyses and few studies of low risk of bias. The strength of our conclusions may thus be limited by those factors. We may uncover only a limited number of comparative studies of transfusion strategies that included a small number of patients. This may potentially limit the planned sensitivity and subgroup analyses. In addition, we will conduct analysis of potential determinants and outcomes associated with transfusion through univariate analysis. In cohort studies, univariate analyses of associations are prone to confusion bias. If sufficient data are available, we will construct a meta-regression, which will help to, at least partially, control for potential confusion.

Little is known on optimal red blood cell transfusion strategies in patients with traumatic brain injury. In order to design studies to improve clinical practices, evidence-based information has to be gathered. We propose to conduct a systematic review that will synthesize the current knowledge from published clinical studies in the field. Our results will be used to optimize future prospective studies on this topic in order to conduct high-quality and rigorous studies with the aim of increasing the quality of care received by patients with traumatic brain injury.

## Abbreviations

CRASH: Corticosteroid randomization after significant head injury; GCS: Glasgow Coma Scale; GOS: Glasgow Outcome Scale; GRADE: Grading of Recommendations Assessment Development and Evaluation; ICU: Intensive care units; IMPACT: International Mission for Prognosis and Analysis of Clinical Trials in TBI; PRISMA: Preferred Reporting Items for Systematic Reviews and Meta-Analyses; RCT: Randomized controlled trial; TRICC: Transfusion Requirements in Critical Care.

## Competing interests

The authors declare that they have no competing interests.

## Authors’ contributions

AB made substantial contributions to conception and design of the review and drafted the manuscript, has given final approval of the version to be published and agreed to be accountable for all aspects of the work. AFT, MC, MS, FL, LM, RZ, JL, DAF and PD contributed to the discussion over the conception and design of the review, revised the manuscript critically for important intellectual content, have given final approval of the version to be published and agree to be accountable for all aspects of the work.

## Supplementary Material

Additional file 1 Search strategy for MEDLINE/PubMed. Click here for file

Additional file 2 Outcome variables. Click here for file
