# Supplementary material for: Red blood cell transfusion in patients with traumatic brain injury: a systematic review protocol
Source: Syst Rev. 2014 Jun 18;3:66. doi: 10.1186/2046-4053-3-66 (PMC4090399; doi:10.1186/2046-4053-3-66)
Supplement: Additional file 1 — Search strategy for MEDLINE/PubMed. [file 2046-4053-3-66-S1.pdf]

Appendix 1. Search strategy for MEDLINE/PubMed

1.
neurocritic*[TIAB] OR
neurointensive[TIAB] OR
neurotrauma*[TIAB] OR
((brain[TIAB] OR
brainstem*[TIAB] OR
head[TIAB] OR
craniocerebral*[TIAB] OR
intracrani*[TIAB] OR
intra-crani*[TIAB] OR
intercrani*[TIAB] OR
inter-crani*[TIAB] OR
cerebr*[TIAB] OR
intracerebral[TIAB] OR
cerebel*[TIAB] OR
forebrain*[TIAB] OR
neurologic*[TIAB] OR
subarachnoid*[TIAB] OR
subdural[TIAB] OR
epidural[TIAB] OR
extradural[TIAB] OR
parenchymal[TIAB] OR
intraparenchymal[TIAB] OR
subcortical[TIAB]) AND
(injury[TIAB] OR
injuries[TIAB] OR
injured[TIAB] OR
trauma[TIAB] OR
traumas[TIAB] OR
traumatic*[TIAB] OR
traumato*[TIAB] OR
damag*[TIAB] OR
contusion*[TIAB] OR
lesion*[TIAB])) OR
TBI[TIAB] OR
skull fracture[TIAB] OR
skull fractures[TIAB] OR
traumatic encephalopath*[TIAB] OR
"diffuse axonal injury"[TIAB] OR
"diffuse axonal injuries"[TIAB] OR
DAI[TIAB] OR
"diffuse axonal damage"[TIAB] OR
"diffuse axonal damages"[TIAB] OR
"Craniocerebral Trauma"[MeSH:NoExp] OR
"Brain Injuries"[MeSH:NoExp] OR
"Diffuse Axonal Injury"[MeSh:NoExp] OR
"Coma, Post-Head Injury"[MeSH:NoExp] OR
"Head Injuries, Closed"[MeSH:NoExp] OR
"Brain Hemorrhage, Traumatic"[MeSH] OR
"Skull Fractures"[Mesh] OR

"Cerebrovascular Trauma" [MeSH]

2.
anemia[TIAB] OR
anaemia[TIAB] OR
anemic[TIAB] OR
anaemic[TIAB] OR
transfusion*[TIAB] OR
rbc therapy [TIAB] OR
rbc therapies [TIAB] OR
rbc management [TIAB] OR
rbc level* [TIAB] OR
rbc target* [TIAB] OR
rbc concentrate*[TIAB] OR
red cell therapy [TIAB] OR
red cell therapies [TIAB] OR
red cell management [TIAB] OR
red cell level*[TIAB] OR
red cell concentrate*[TIAB] OR
red cells therapy [TIAB] OR
red cells therapies [TIAB] OR
red cells management [TIAB] OR
red cells level*[TIAB] OR
red cells concentrate*[TIAB] OR
red blood cell therapy[TIAB] OR
red blood cell therapies[TIAB] OR
red blood cell management[TIAB] OR
red blood cell level*[TIAB] OR
red blood cell concentrate*[TIAB] OR
red blood cells therapy[TIAB] OR
red blood cells therapies[TIAB] OR
red blood cells management[TIAB] OR
red blood cells level*[TIAB] OR
red blood cell concentrate*[TIAB] OR
blood therapy[TIAB] OR
blood therapies[TIAB] OR
blood management[TIAB] OR
erythrocyte therapy[TIAB] OR
erythrocyte therapies[TIAB] OR
erythrocyte management[TIAB] OR
erythrocyte level*[TIAB] OR
erythrocyte concentrate*[TIAB] OR
erythrocytes therapy[TIAB] OR
erythrocytes therapies[TIAB] OR
erythrocytes management[TIAB] OR
erythrocytes level*[TIAB] OR
erythrocytes concentrate*[TIAB] OR
hb trigger*[TIAB] OR
hb threshold*[TIAB] OR
hb management[TIAB] OR
hb level*[TIAB] OR
hb target*[TIAB] OR

haemoglobin trigger*[TIAB] OR
haemoglobin threshold*[TIAB] OR
haemoglobin management[TIAB] OR
haemoglobin level*[TIAB] OR
haemoglobin target*[TIAB] OR
hemoglobin trigger*[TIAB] OR
hemoglobin threshold*[TIAB] OR
hemoglobin management[TIAB] OR
hemoglobin level*[TIAB] OR
hemoglobin target*[TIAB] OR
hct trigger*[TIAB] OR
hct threshold*[TIAB] OR
hct management[TIAB] OR
hct level*[TIAB] OR
hct target*[TIAB] OR
haematocrit trigger*[TIAB] OR
haematocrit threshold*[TIAB] OR
haematocrit management[TIAB] OR
haematocrit level*[TIAB] OR
haematocrit target*[TIAB] OR
hematocrit trigger*[TIAB] OR
hematocrit threshold*[TIAB] OR
hematocrit management[TIAB] OR
hematocrit level*[TIAB] OR
hematocrit target*[TIAB] OR
Blood Transfusion [MeSh]

3.
Animals[MeSH] NOT Humans[MeSH]

4.
#1 AND #2 NOT #3
