# Supplementary material for: Red blood cell transfusion in patients with traumatic brain injury: a systematic review protocol
Source: Syst Rev. 2014 Jun 18;3:66. doi: 10.1186/2046-4053-3-66 (PMC4090399; doi:10.1186/2046-4053-3-66)
Supplement: Additional file 2 — Outcome variables. [file 2046-4053-3-66-S2.pdf]

Appendix 2. Outcome variables

**Outcomes**					**NS**
	**Timing of assessment**	**Reported effect size, adjusted* (95%CI; p-value)**	**Transfused (n=)**	**Not transfused (n=)**	
Mortality (n)					
WLST (n)					
GOS (ct)					
GOSe (ct)					
Poor neurological outcome, GOS < 4 (n)					
Poor neurological outcome, GOSe < 5 (n)					
Length of ICU stay					
Length of hospital stay					
Duration of mechanical ventilation					
Other relevant clinical outcome					
**Complications / adverse events**<br>*(please specify in the first column : if they can be attributable to RBC transfusion)*					
Re-bleeding (n)					
MODS (n)<br>*Multiple organ dysfunction syndrome*					
SOFA					
Cerebral infarction (n)					
Myocardial infarction (n)					
Vasospasm (n)					
DIC (n)<br>*Disseminated intravascular coagulation*					
DVT (n)<br>*Deep venous thrombosis*					
Thrombo-embolism (n)					
Volume overload / Pulmonary oedema (n)					
ARDS (n)<br>*Acute respiratory distress syndrome*					
ALI (n)<br>*Acute lung injury*					
TRALI (n)<br>*Transfusion related acute lung injury*					
Infection (n) (non-VAP)					
VAP<br>*Ventilator-associated pneumonia*					
Other (specify)					
